# Identification and Functional Characteristics of *NR5A1* Gene Variant in Patients with 46,XY Disorders of Sex Development

**DOI:** 10.3390/genes17091070

**Published:** 2026-09-04

**Authors:** Lin He, Liangzhe Li, Yuxiao Li, Yujun Sun, Zhi Zheng, Chunfang Chu, Shuya Chen, Lin Li

**Affiliations:** 1 Central Laboratory, Beijing Obstetrics and Gynecology Hospital, Capital Medical University, Beijing Maternal and Child Health Care Hospital, Beijing 100006, China; 2 Department of Gynecology, Beijing Obstetrics and Gynecology Hospital, Capital Medical University, Beijing Maternal and Child Health Care Hospital, Beijing 100026, China; chuchunfang@ccmu.edu.cn; 3 Department of Gynecological Oncology, Beijing Obstetrics and Gynecology Hospital, Capital Medical University, Beijing Maternal and Child Health Care Hospital, Beijing 100006, China

**Keywords:** 46,XY disorders of sex development, NR5A1, whole-exome sequencing, functional analysis, RNA sequencing

## Abstract

Background/Objectives: Individuals with 46,XY disorders of sexual development (DSD) present with incomplete genital masculinization, aberrant gonadal development, and occasional retention of Müllerian duct remnants. Genetic factors play a substantial role in DSD pathogenesis, and whole-exome sequencing has expanded the catalog of candidate variants in recent years. However, the underlying molecular pathways remain incompletely characterized, and the functional relevance of most isolated genetic findings has not been systematically determined. This study aimed to identify and functionally characterize novel *NR5A1* variants in DSD. Methods: A heterozygous missense variant *NR5A1* c.88T>A (p.Cys30Ser) was identified in two patients with DSD, and initial functional characterization of the variant was performed via immunofluorescence analysis, Western Blotting, RNA sequencing, and quantitative real-time PCR analysis. Results: Wildtype NR5A1 protein localized predominantly to the nucleus, whereas the p.Cys30Ser mutant exhibited dual nuclear and cytoplasmic distribution. Compared with the wildtype, the p.Cys30Ser variant altered the expression of 642 genes, with differentially expressed genes primarily enriched in the neuroactive ligand–receptor interaction pathway. The variant impaired the transactivation of canonical *NR5A1* downstream targets, resulting in the marked downregulation of 560 genes including key regulators such as *KISS1R*, *CYP11A1*, *STAR*, *GABRP*, and *GRAMD1D*. Conclusions: This study is the first to identify and functionally characterize the *NR5A1* p.Cys30Ser variant in the context of DSD. Our findings broaden the mutational spectrum of *NR5A1*-related DSD and provide new insights into the molecular genetic basis of sexual development disorders.

## 1. Introduction

46,XY disorders of sex development (DSD) (OMIM #612965) (ORPHA:98085) are characterized by a wide phenotypic spectrum, ranging from under-virilized external genitalia and gonadal dysgenesis to the persistence of Müllerian derivatives. The past decade has witnessed an exponential increase in genetic discoveries, largely driven by whole-exome sequencing, yet the functional interpretation of most identified variants remains elusive. Many candidate genes have been catalogued, but their causative roles and downstream molecular cascades are still poorly defined, leaving a substantial gap between genotype and pathophysiology.

The establishment of bipotential gonads and their subsequent differentiation into testes or ovaries depend on an intricate regulatory circuitry involving transcription factors, steroidogenic enzymes, and intercellular signaling molecules. Disruption of any nodal point in this cascade can give rise to DSD, which accordingly presents with heterogeneous clinical features. Among the key players, NR5A1 (also known as steroidogenic factor 1, SF-1, or Ad4BP) has emerged as a critical regulator of adrenal and gonadal development, as well as of hypothalamic–pituitary axis function. Encoded by *NR5A1* on chromosome 9q33.3, this nuclear receptor comprises a conserved DNA-binding domain (DBD) with two zinc fingers, a hinge region, a ligand-binding domain (LBD), and two activation function domains (AF-1 and AF-2) [1]. The protein demonstrates significant evolutionary conservation, with 95% amino acid identity between mice and humans [2]. The expression of NR5A1 initiates in the urogenital ridge and later broadens to encompass tissues such as the ventral hypothalamus, pituitary, adrenals, and gonads. NR5A1 is expressed in Leydig and Sertoli cells throughout testicular development [3]. In mice, Nr5a1 is found in steroidogenic cortical cells, where it is expressed continuously from embryogenesis into postnatal life [4].

The functional scope of NR5A1 spans from fundamental developmental processes of reproduction and development—such as adrenal and gonadal formation, sex determination, and ventromedial hypothalamic nucleus (VMH) establishment—to the ongoing regulation of endocrine physiology. It also fulfills its role by serving as a key transcriptional activator for numerous genes within the reproductive and steroidogenic pathways (e.g., AMH, ACTH, HMG-CoA synthase, steroidogenic acute regulatory protein (STAR), 3β-hydroxysteroid dehydrogenase (3βHSD), and several cytochrome P450 steroid hydroxylase (CYP) enzymes [5]), thereby ensuring the integrated hormone signaling essential for reproduction [6]. WT1 [7] and LHX9 regulate initial gonadal development through NR5A1 [8]. In fetal Sertoli cells, NR5A1 upregulates the transcription of SOX9, thereby regulating the development of Sertoli cells and the expression of AMH [9,10]. The functional loss mutation of NR5A1 is associated with a wide range of DSD [5,11]. Since the first NR5A1 mutation (revealed a heterozygous 2 bp G35E (GGC→GAA) mutation in exon 3) was described in DSD in 1999 [6], the NR5A1-related phenotypes have substantially widened, including 46,XY complete and partial gonadal dysgenesis, penoscrotal hypospadias (c.319C>T, c.31G>T, etc.), microphallus with anorchidia (V355M) and 46,XX primary ovarian insufficiency (POI) (c.877G>A, c.3G>A, etc. [12,13,14,15]). Functional studies in animal models have highlighted the non-redundant role of NR5A1: targeted Nr5a1 ablation in mice leads to adrenal and gonadal agenesis, male-specific Müllerian duct persistence, and hypothalamic–pituitary defects [16,17,18]. Furthermore, most of the published studies were case reports; the pathogenic mechanisms underlying these mutations and their link to diseases remain to be unraveled.

This study aimed to explore the genetic etiology of DSD. Through whole-exome sequencing (WES), we identified a NR5A1 p.C30S (ClinVar Accession ID: VCV004082474.2) variant in patients diagnosed as 46,XY DSD and investigated its correlation. Our findings provide important and novel insights into the pathophysiology of this condition and offer a valuable reference for further genetic diagnosis and research.

## 2. Materials and Methods

### 2.1. Patients and Ethical Approval

Two patients diagnosed with DSD at the Beijing Obstetrics and Gynecology Hospital, Capital Medical University were included in our study. Their chromosome karyotypes were confirmed to be 46,XY. The research protocol received approval from the Ethics Committees of Beijing Obstetrics and Gynecology Hospital, Capital Medical University (2021-KY-032-01), in compliance with the ethical principles set forth in the Declaration of Helsinki [1964] and its subsequent revisions.

### 2.2. WES, In Silico Analysis and Sanger Sequencing

Genomic DNA extraction, whole-exome sequencing (WES), and Sanger sequencing were performed following a previously described protocol [19]. Sanger sequencing was carried out with locus-specific primers (listed in Table A1) to verify the variant identified by WES. Ultimately, two patients carrying NR5A1 mutations were identified for further analysis.

### 2.3. 293FT Cell Culture, Plasmid Construction and Transfection

293FT cells were maintained under standard conditions as described elsewhere, and transient transfections were executed using jetPRIME reagent (Polyplus, Illkirch-Graffenstaden, France, Cat# 101000046) following the manufacturer’s instructions [20].

Wildtype (WT, NM_004959.5) pcDNA3.1(+)-CMV-NR5A1-3 × Flag were purchased commercially from Ruibiotech (Beijing, China) and mutant (c.88T>A; p.C30S) human NR5A1 overexpression plasmids were constructed by Ruibiotech (Beijing, China), with the empty pcDNA3.1(+) vector used as a control.

### 2.4. Antibodies

The following primary antibodies were used: anti-GAPDH (60004-1-Ig; ProteinTech, Wuhan, China) and anti-DYKDDDDK tag (66008-4-Ig; ProteinTech, Wuhan, China). Secondary antibodies included a biotinylated anti-mouse IgG (H + L) (ZB-2305/ZB-2301; ZSGB-BIO, Beijing, China) and an Alexa Fluor™ 488-conjugated anti-mouse IgG (H + L) cross-adsorbed secondary antibody (A-11005; Invitrogen, Carlsbad, CA, USA). All antibodies were diluted according to standard protocols.

### 2.5. RNA Extraction, Reverse Transcription, and Real-Time Quantitative Polymerase Chain Reaction (qPCR)

RNA extraction, cDNA synthesis, and quantitative real-time PCR (qPCR) assays were performed following a previously described protocol [21]. Gene expression levels were normalized against the endogenous control ACTB and analyzed via the 2^−ΔΔCt^ method for relative quantification. All primer sequences are provided in Table A2.

### 2.6. RNA Sequencing (RNA-seq) and Data Analysis

RNA sequencing was conducted by Novogene (Beijing, China). RNA-seq and data analysis were performed as described previously [22].

### 2.7. Western Immunoblotting and Immunofluorescence Staining

Following transfection with NR5A1 constructs, 293FT cells were processed for immunofluorescence 48h post-transfection. The methodology was described in detail in our previous study [21].

### 2.8. Statistical Analysis

All statistical analyses were performed as previously described [19].

## 3. Results

### 3.1. Clinical Spectrum of the Identified DSD Patients Harboring NR5A1 Variant

**Figure 1:**
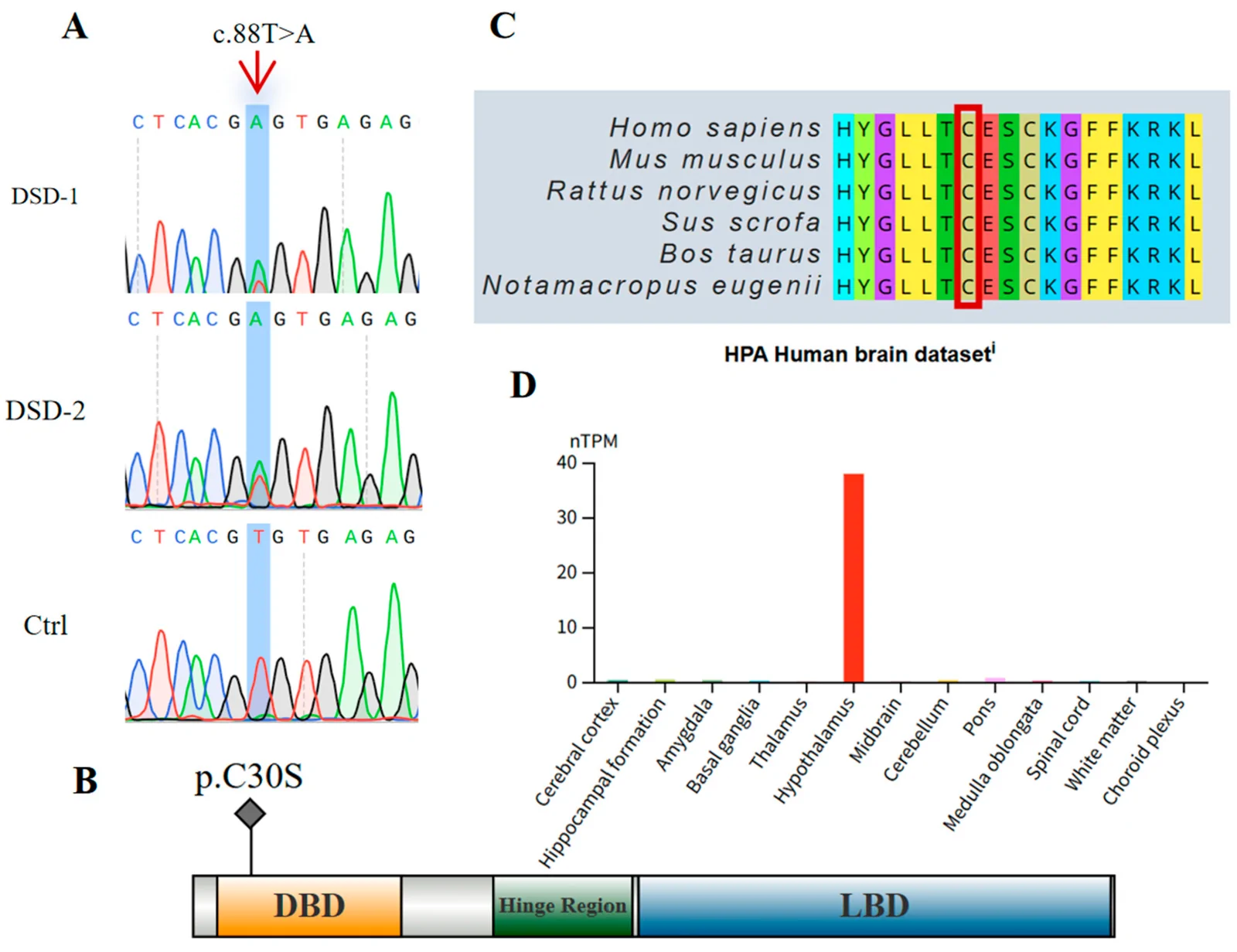
Genetic analysis of the *NR5A1* variant. (**A**) Sanger sequencing validated in patient DSD-1&2. The red arrow indicated the mutant site (c.88T>A). (**B**) The scheme for NR5A1 protein structures with the variant indicated with its position. DBD, DNA-Binding Domain; LBD, Ligand-Binding Domain. (**C**) The amino acid conservancy in the region of NR5A1 surrounding codon 30. The affected residue (C30S) was marked by red rectangle. The different colors represent different amino acids. (**D**) Gene expression in brain for NR5A1. The data was obtained from an online database https://www.proteinatlas.org/ENSG00000136931-NR5A1/brain (accessed on 15 November 2025).

Two Chinese Han patients with DSD were included. According to the results of WES, two patients (DSD-1&DSD-2) carried a heterozygous missense variant in *NR5A1* (NM_004959.5), c.88T>A; p.Cys30Ser. The clinical profile of patient DSD-1 and DSD-2 is summarized in Table 1. Both patients present with 46,XY DSD, primary amenorrhea, absent Müllerian structures, and hypergonadotropic hypogonadism, though DSD-2 exhibits signs of virilization and confirmed testicular tissue. Sanger sequencing confirmed the presence of the variant (Figure 1A), which was located within the DNA-binding domain of NR5A1 (Figure 1B). The affected residue (C30S) was highly conserved across species (Figure 1C), and given the strong expression of NR5A1 in the human hypothalamus (Figure 1D, The Human Protein Atlas), we proceeded with functional assays to evaluate its pathogenic potential. Detailed clinical information and *in silico* prediction results are summarized in Table 2.

### 3.2. C30S Variant Alters Normal RNA and Protein Expression and Subcellular Localization

**Figure 2:**
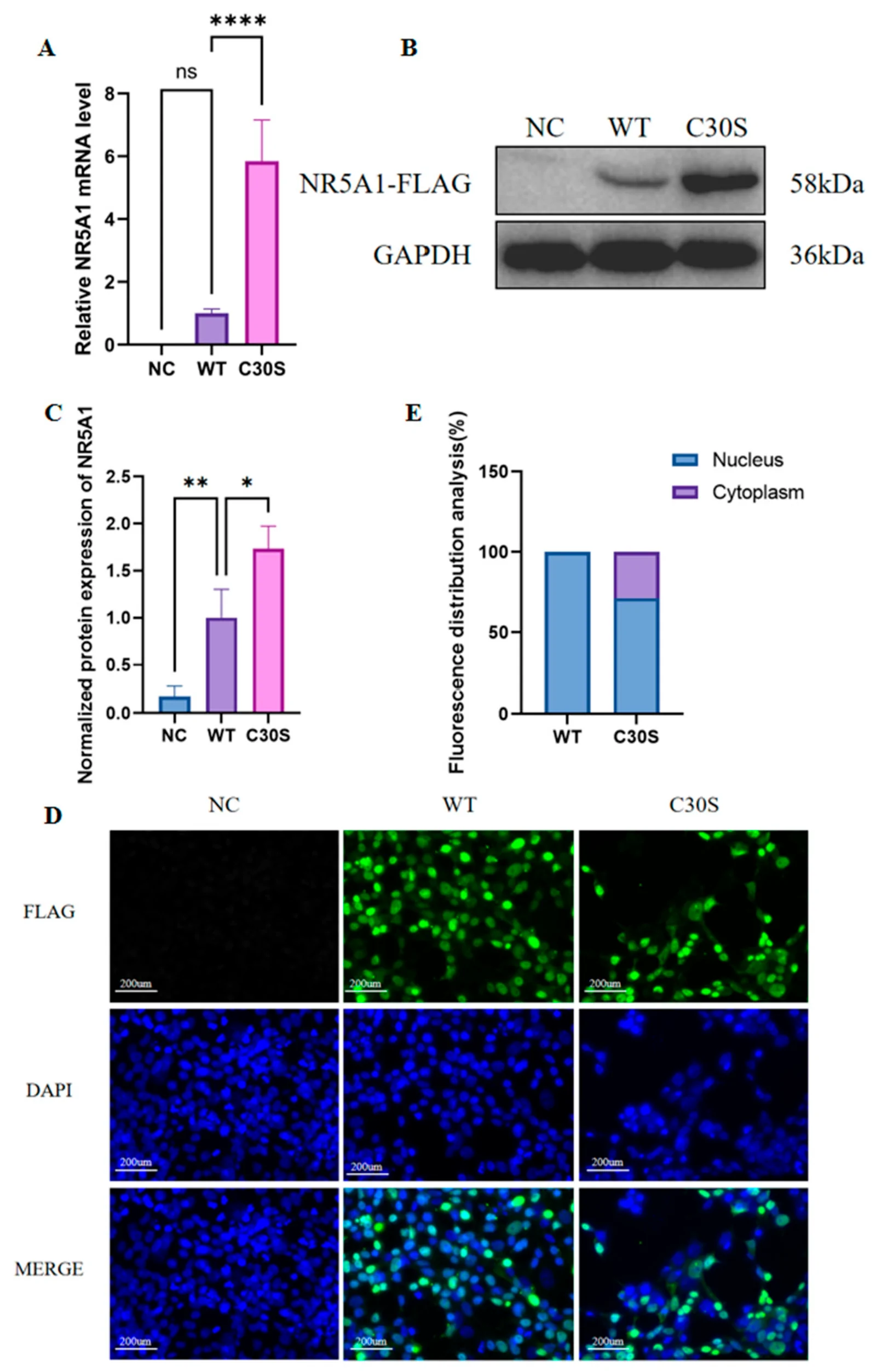
Functional analysis of the pathogenicity of human *NR5A1* variant carried out in 293FT cells after transfection with WT or mutant *NR5A1* plasmids. (**A**) Reverse transcription polymerase chain reaction analyses of *NR5A1* mRNA level. *ACTB* was used as the reference gene. NC, negative control; WT, wild-type; ns, not significant. (**B**) Western blot analysis of NR5A1 (Nuclear Receptor Subfamily 5 Group A Member 1) expression. GAPDH (Glyceraldehyde-3-Phosphate Dehydrogenase) was used as the loading control. (**C**) Normalized protein expression of NR5A1. Densitometric quantification of three independent experiments (normalized to GAPDH) revealed that the C30S mutant significantly upregulated NR5A1 protein levels compared with the WT group (* *p* < 0.05, ** *p* < 0.01, one-way ANOVA followed by Dunnett’s test). The corresponding quantitative data are shown in the bar graph (mean ± SD). (**D**) Immunofluorescence staining of WT and mutant NR5A1 proteins. The cellular nucleus was visualized using DAPI (4′,6-Diamidino-2-Phenylindole) (blue). The flag-tagged protein was stained in green. (**E**) Fluorescence distribution analysis of nucleus/cytoplasm of WT and mutant NR5A1 protein. In A, data are presented as the mean ± SD of 3 independent experiments. **** *p* < 0.0001, one-way ANOVA followed by Dunnett’s test.

To evaluate the functional impact of the NR5A1 variant, we performed established in vitro assays. qPCR analysis showed an increase in the *NR5A1* mRNA expression level in the C30S mutant compared to the wildtype (WT) (Figure 2A). Western blot analysis revealed a significant increase in NR5A1 protein expression in the C30S mutant compared with WT (Figure 2B,C). Immunofluorescence staining revealed that the C30S variant largely retained proper nuclear localization, similar to the exclusively nuclear WT protein, although 30–35% cytoplasmic presence was detected in a fraction of cells (Figure 2D,E). Collectively, these results indicate that while the C30S variant does not significantly affect NR5A1 protein stability, it disrupts its normal subcellular localization. However, the data presented in this section do not permit definitive conclusions as to whether the C30S variant altered the transcriptional activation activity of the NR5A1 protein.

### 3.3. RNA-Seq Analysis Revealed a Loss of Regulatory Function in the NR5A1 C30S Variant

**Figure 3:**
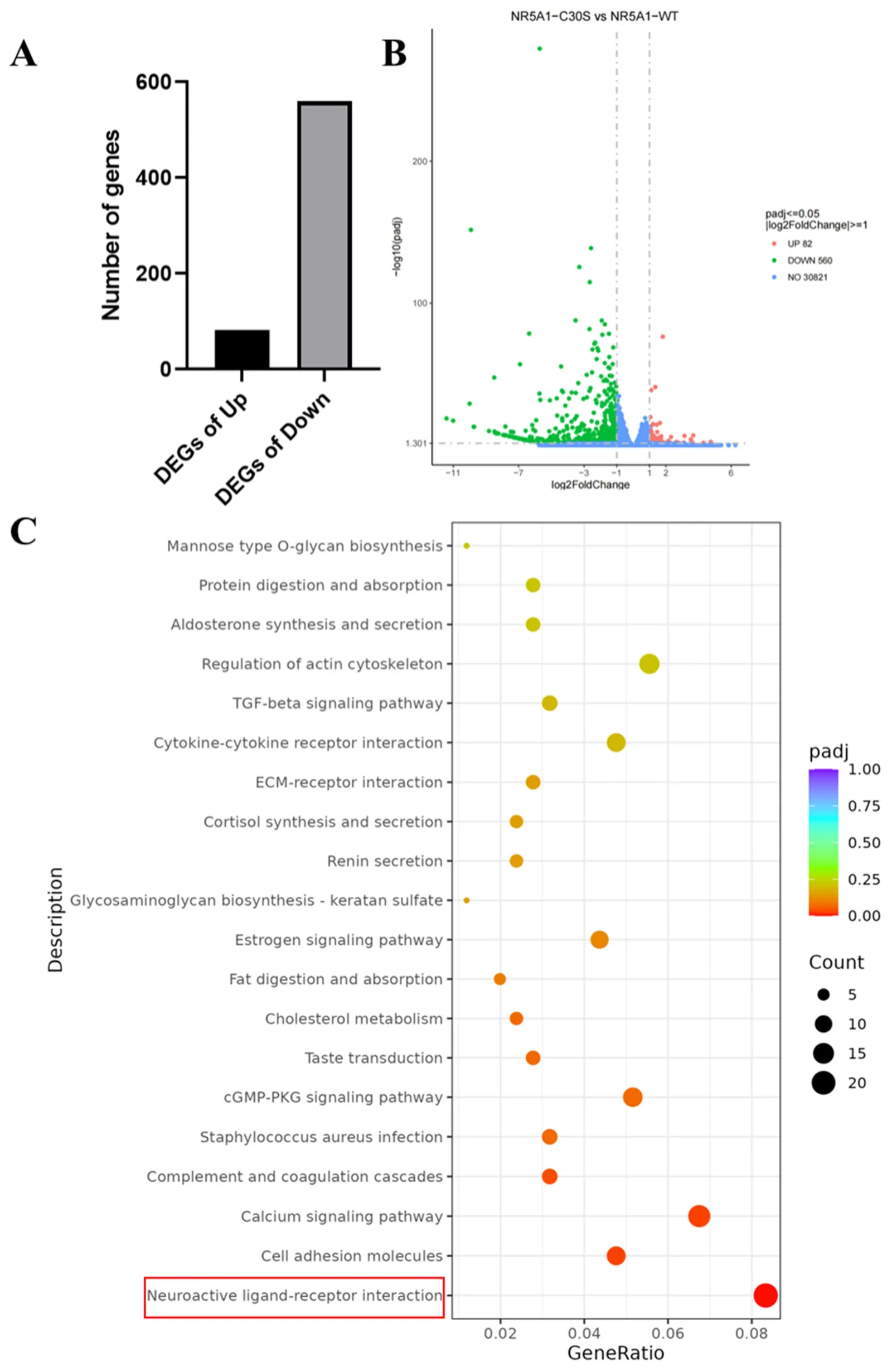
The transcriptome sequencing result of RNA extracted from 293FT cells after transfection with WT and mutant *NR5A1* overexpression plasmids. (**A**) Bar chart showing the number of differentially expressed genes (DEGs) identified by RNA-sequencing analysis between NR5A1-C30S mutant and NR5A1-WT (wild-type) groups. The *x*-axis indicates the direction of expression change, with “DEGs of Up” representing significantly upregulated genes and “DEGs of Down” representing significantly downregulated genes. The *y*-axis represents the total number of genes in each category. Genes were classified as differentially expressed based on the thresholds of adjusted *p* value (padj) ≤ 0.05 and |log2(fold change)| ≥ 1. A total of 82 genes were significantly upregulated (black bar) and 560 genes were significantly downregulated (gray bar) in the NR5A1-C30S group compared with the NR5A1-WT group. (**B**) Volcano plot of DEGs between NR5A1-C30S and NR5A1-WT. Each point represents a gene; red points indicate significantly upregulated genes (n = 82), green points indicate significantly downregulated genes (n = 560), and blue points indicate genes with no significant change (n = 30,821). Significance was defined as padj ≤ 0.05 and |log2(fold change)| ≥ 1. Dashed lines indicate the corresponding thresholds: vertical dashed lines denote |log2(fold change)| = 1, and the horizontal dashed line denotes padj = 0.05 (−log10(padj) = 1.301). (**C**) KEGG pathway enrichment analysis of DEGs. The *x*-axis represents the gene ratio (proportion of DEGs annotated to each pathway), and the *y*-axis lists the enriched KEGG pathways. Point size indicates the number of enriched genes (Count), and point color represents the adjusted *p* value (padj), with red indicating higher significance. The red box highlights the “Neuroactive ligand-receptor interaction” pathway, which shows the highest gene ratio, the largest gene count, and the lowest padj among all enriched terms, representing the most significantly enriched pathway in this analysis.

**Figure 4:**
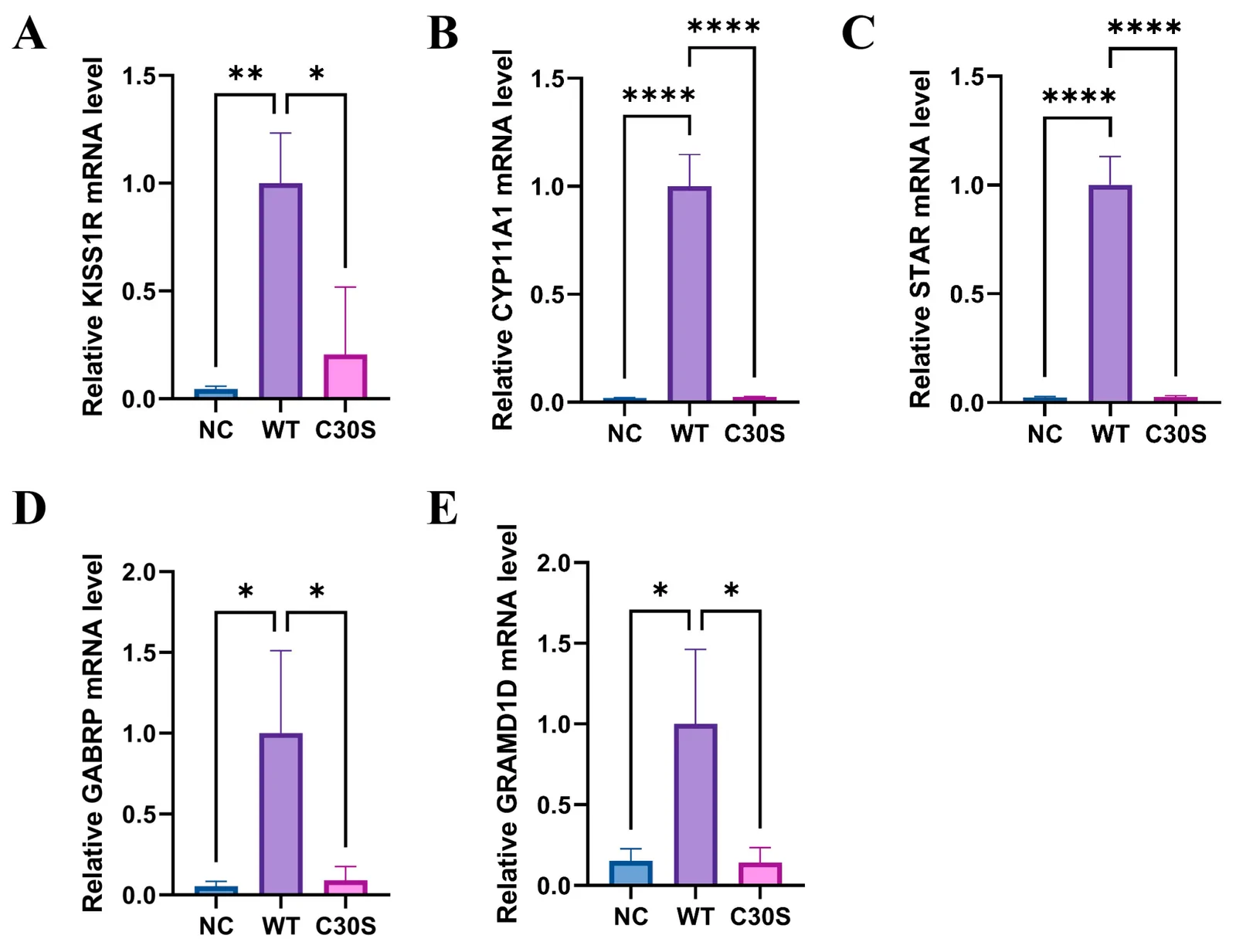
(**A**–**E**) Quantification of mRNA expression of several NR5A1-regulated DEGs detected by RT-qPCR. *ACTB* was used as the reference gene. Data were presented as the mean ± SD of 3 independent experiments. **** *p* < 0.0001, ** *p* < 0.01, * *p* < 0.05; ns—not significant; one-way ANOVA followed by Dunnett’s test.

**Figure A1:**
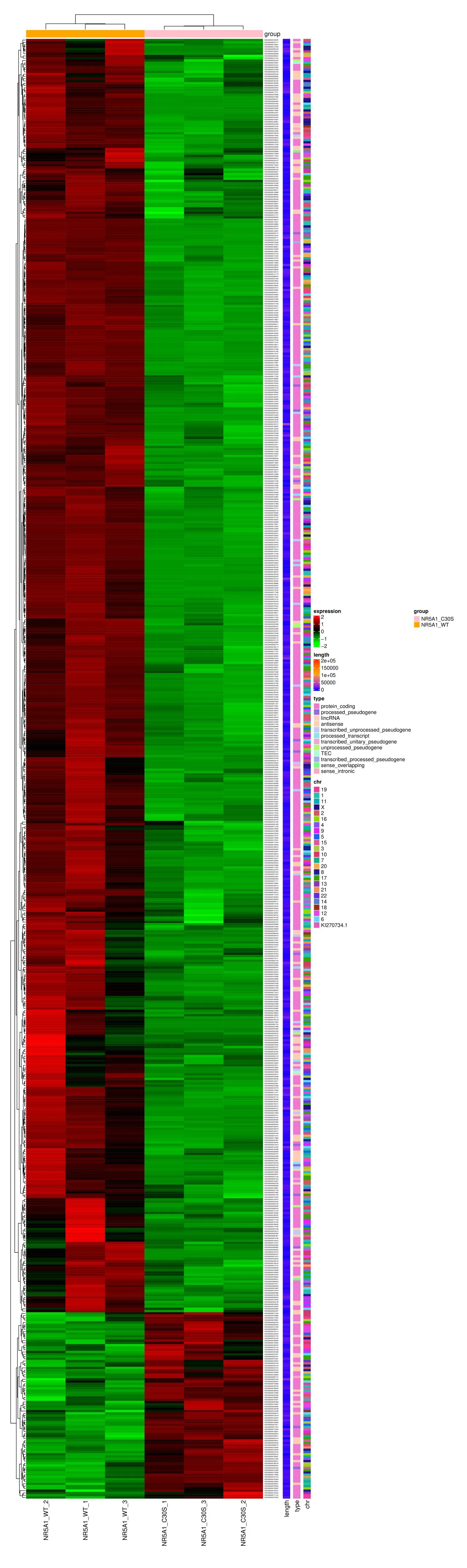
The heatmap of DEGs among WT and mutant group.

To elucidate the molecular pathogenesis of the C30S variant in DSD, we assessed its impact on the transactivation function of NR5A1, a key transcriptional regulator. RNA sequencing in 293FT cells overexpressing either the wild-type (WT) or C30S variant identified 642 differentially expressed genes (DEGs), with a pronounced downregulation (560 genes) in the C30S group (Figure 3A). The DEGs were depicted in a volcano plot (Figure 3B) and a heatmap (Figure A1). Kyoto Encyclopedia of Genes and Genomes (KEGG) pathway analysis revealed that the downregulated genes were significantly enriched in key processes, including “neuroactive ligand–receptor interaction”, “cGMP-PKG signaling pathway”, and “complement and coagulation cascades” (Figure 3C). We independently validated a subset of markedly downregulated genes (CYP11A1, GABRP, KISS1R, STAR, and GRAMD1D) using q-PCR (Figure 4).

Notably, expression of KISS1R and GABRP—both implicated in the “neuroactive ligand–receptor interaction” pathway—was substantially suppressed, suggesting that NR5A1 may contribute to DSD by disrupting the regulation of genes critical to the hypothalamic–pituitary–gonadal/adrenal (HPG/HPA) axes [23]. Furthermore, the C30S variant also altered the expression of genes implicated in steroid hormone synthesis and secretion, such as *CYP11A1* and *STAR*, as well as cholesterol trafficking gene (*GRAMD1D*) [24].

## 4. Discussion

Sex development is an exceedingly complex process that requires the coordinated action of various hormones, transcription factors, and signaling molecules. During the fourth to fifth embryonic week, primordial germ cells relocate to the genital ridges, establishing the bipotential gonad [25,26]. This process is regulated by multiple genes such as *EMX2, CBX2, BMP4, NR5A1*, and *WT1* [7]. If the primordial germ cells that migrate into the genital ridge carry XY sex chromosomes, the sex-determining gene SRY in the sex-determining region on the Y chromosome is expressed. Together with NR5A1, it activates the expression of the downstream gene *SOX9*, directing the gonad to differentiate into a testis. If the primordial germ cells contain XX chromosomes, the gonad naturally develops into an ovary, a process regulated by genes including *FOXL2, RSPO1, WNT4*, and *CTNNB1* [27]. Sex determination is the process by which chromosomal sex directs the differentiation of the bipotential gonad into either a testis or an ovary.

*NR5A1* mutation is a common cause of 46,XY DSD, with an incidence rate of approximately 10–20% [28,29]. NR5A1 is a key transcription factor regulating the synthesis of various steroid hormones and the expression of genes related to sexual development, affecting the hypothalamic–pituitary–gonadal/adrenal (HPG/HPA) axes [17,30]. The mutation C30S is located in the highly conserved DNA binding domain, playing an important role in identifying and binding DNA elements of target genes, which are essential for mediating DNA and zinc binding [31]. Consequently, genetic mutations are thought to interfere with these interactions, which in turn suppresses transcriptional activation and leads to a spectrum of NR5A1 functional deficiencies [9]. Our findings reinforce the critical role of NR5A1 in DSD. Given its fundamental position in the genetic regulatory network, NR5A1 dysregulation can disrupt multiple steroidogenic pathways. To functionally characterize a disease-associated variant, we compared the transcriptional profiles of the WT and the C30S group. RNA-seq identified 642 DEGs. Interestingly, compared with the WT group, the expression pattern of *CYP11A1, GABRP, KISS1R, STAR* and *GRAMD1D* was downregulated in the C30S variant group.

A key function of kisspeptin signaling through its G protein-coupled receptor (KISS1R) is to regulate the hypothalamic–pituitary–gonadal (HPG) axis, thereby playing a pivotal role in mammalian reproductive control [32,33]. DSD patients usually have abnormalities in the HPA axis, such as elevated FSH and LH levels. Male patients with *KISS1R* loss-of-function mutations exhibit gonadotropin resistance, resulting in congenital hypogonadotropic hypogonadism (CHH) [34]. Research has demonstrated that reduced *KISS1R* expression leads to decreased expression of the luteinizing hormone/choriogonadotropin receptor (LHCGR) and the steroidogenic gene *STAR*, thereby contributing to the observed gonadotropin insensitivity [35]. NR5A1 may be involved in the HPA axis regulation and steroid hormone secretion in DSD patients in combination with these factors. However, the specific mechanism remains unclear, possibly through regulation by binding to NR5A1. The mutation of C30S is located in DBD and may be related to interaction of these factors.

Meanwhile, the C30S variant may lead to reduced steroidogenesis, gonadotropin insensitivity and abnormal gonadal development by downregulating the expression of steroidogenic genes *CYP11A1* and *STAR*. The C30S mutation may impair steroid hormone transport by downregulating *GRAMD1D*, a GRAM-domain gene with a putative role in cholesterol trafficking, resulting DSD [36,37].

Given that the gamma-aminobutyric acid type A receptor subunit pi (GABRP) is implicated in breast cancer metastasis [38], primarily through its role in regulating cell migration, and considering that genital tract development also relies on cell migration processes such as epithelial–mesenchymal transition (EMT) [39], we hypothesize that the C30S mutation may impair genital tract development by downregulating GABRP expression and consequently disrupting cellular migration.

At the same time, this mutation has been reported by LaPiscina et al. [9] and the patient also had *AMH* and *STAR* variants, but the clinical phenotype was different from the patients in this study. At birth, the patient reported by LaPiscina et al. had a curved small penis, hypospadias, and undescended testes, which required surgery. Later MRI results showed a normal penis and testes but no Müllerian ducts. For the two patients in this study, one had an absence of female reproductive organs but no male reproductive organs, while the other had both male and female reproductive systems. The reason might be that the genetic pattern of the disease could have incomplete penetrance and may be polygenic. Meanwhile, the luciferase reporter assay result in LaPiscina et al. [9] also supports our research findings.

The primary constraint of the research is the modest number of DSD cases examined. To advance our understanding of disease pathogenesis and confirm inheritance modes, it will be necessary to assemble larger patient cohorts, incorporate detailed family pedigrees, and leverage functional studies. The promising prospects offered by advancing sequencing technologies will significantly aid in discovering new variants governing DSDs, ultimately leading to a more complete etiological understanding.

## 5. Conclusions

Elucidating the genetic architecture underlying DSDs remains a fundamental priority in reproductive genetics. Collectively, our findings not only expand the known mutational spectrum of *NR5A1* but also offer mechanistic insights into its pivotal role in sex development, thereby facilitating a deeper comprehension of its pathogenic cascades.

## Figures and Tables

**Table 1:** Clinical profile of the patients with *NR5A1* variant.

	DSD-1	DSD-2
History	-Family history: primary amenorrhea (Women who are over 15 years old or whose secondary sexual characteristics have matured for more than 2 years but still do not have menstruation) -Karyotype: 46,XY karyotype. The patient was assigned as female.	-Family history: primary amenorrhea -Karyotype: 46,XY karyotype. She is the sister of DSD-1. The patient was assigned as female.
Phenotype	-Secondary Sexual Characteristics: Absent breast development (Tanner stage 1). -External Genitalia: Infantile with sparse pubic hair. -Vagina: Blind-ending pouch; a depth of approximately 0.8 cm was confirmed on probing. -Pelvic Structures: No uterus or adnexal structures were palpable.	-Secondary Sexual Characteristics: Facial hirsutism; absent breast development (Tanner stage 1). -Inguinal Examination: A 2cm diameter, palpable mass medial to the right inguinal canal. -External Genitalia (Female phenotype): -Labia minora: slightly small. -Clitoris: hypertrophied (~1.5 cm long). -Labia majora and mons pubis: no masses palpable. -Hymen: intact. -Vagina: patent, 8 cm in depth. -Laparoscopic exploration revealed the absence of the uterus and bilateral adnexa within the pelvis. Gonadal tissues were identified within both inguinal canals, with the right being slightly larger. -Histopathological examination of the resected gonadal tissues confirmed the presence of testicular tissue.
Investigations	-Abdominal ultrasound (July 2023) showed no uterine contour and no ovarian echo in the bilateral adnexa area. -Hormone levels (July 2023) were as follows: luteinizing hormone (LH): 16.43 IU/L; follicle-stimulating hormone (FSH): 78.33 IU/L; serum testosterone (T): 28.86 ng/dL; estradiol (E2): 11.03 pg/mL; progesterone (P4): 0.17 ng/mL, and prolactin (PRL): 17.06 ng/mL.	-Abdominal ultrasound (July 2023) showed two intra-abdominal gonads (right gonad 0.85 cm and left gonad 0.95 cm in length) and no Müllerian duct remnants. -Hormone levels (July 2023) were as follows: LH: 19.50 IU/L; FSH: 61.67 IU/L; T: 284,04 ng/dL; E2: 0.10 pg/mL; P4: 0.81 ng/mL; and PRL: 24.68 ng/mL.

**Table 2:** Baseline information and in silico analysis of the NR5A1 variant identified in the patients with DSD.

Parameter	Result
Mutation type	Missense
Exon	2
Variant	c.88T>A
Amino acids	p.C30S
Inheritance	Heterozygous
GnomAD/ExAC frequencyA	0
Algorithms	
AlphaMissense	Likely_pathogenic
CADD	28.4 (Likely deleterious)
SIFT	0.00 (Affect protein function)
PolyPhen-2	0.990 (Possibly Damaging)

CADD, Combined Annotation Dependent Depletion.

## Data Availability

The WES data are available from the corresponding author upon reasonable request. Other data generated or analyzed during the study are included in this published article.

## Abbreviations

DSD: disorders of sex development; NR5A1: nuclear receptor subfamily 5 group A member 1; SF-1: steroidogenic factor 1; WES: whole-exome sequencing; RNA-seq: RNA sequencing; DEGs: differentially expressed genes; KEGG: Kyoto Encyclopedia of Genes and Genomes; HPG: hypothalamic–pituitary–gonadal; HPA: hypothalamic–pituitary–adrenal; STAR: steroidogenic acute regulatory protein; qPCR: quantitative real-time polymerase chain reaction.

## Appendix A

**Table A1:** Primers used for Sanger sequencing of patients with *NR5A1* variant.

Primer Name	Sequence (5′→3′)
p.C30S-F	ACCCTCATCCGGTGTGAGAG
p.C30S-R	CTGTCTCCAGCTTGAAGCCATT

**Table A2:** Primers used in RT-qPCR.

Primer Name	Sequence (5′→3′)
qPCR-ACTB-F	GCACAGAGCCTCGCCTT
qPCR-ACTB-R	GTTGTCGACGACGAGCG
qPCR-NR5A1-F	CCGGCTACCACTACGGACT
qPCR-NR5A1-R	CCGCAGGGCTAGGATTTATTG
qPCR-CYP11A1-F	GCAGTGTCTCGGGACTTCG
qPCR-CYP11A1-R	GGCAAAGCGGAACAGGTCA
qPCR-KISS1R-F	CAGCGCTTCCATTGTCAGAA
qPCR-KISS1R-R	CTCCTTGTCGCCTGCATATG
qPCR-STAR-F	GAGAAGTCTTGCTTTATGGGCTCAAGAATG
qPCR-STAR-R	GGTGCCTATGAAAGCAATAGGGAAACATGT
qPCR-GABRP-F	CTTGGCCTTCGTGTGTCTGAG
qPCR-GABRP-R	CCGACCTCGACGTTGAACT
qPCR-GRAMD1D-F	AGAAGGGCTCAGATCACTCCT
qPCR-GRAMD1D-R	GTGGGGCTTAACACATTATACCA
